# Compare with different vegetable oils on the quality of the *Nemipterus virgatus* surimi gel

**DOI:** 10.1002/fsn3.2889

**Published:** 2022-06-02

**Authors:** Chunyong Song, Yufeng Lin, Pengzhi Hong, Huanming Liu, Chunxia Zhou

**Affiliations:** ^1^ College of Food Science and Technology Guangdong Provincial Key Laboratory of Aquatic Product Processing and Safety Guangdong Provincial Engineering Technology Research Center of Marine Food Guangdong Modern Agricultural Science and Technology Innovation Center Guangdong Ocean University Zhanjiang Guangdong China; ^2^ Southern Marine Science and Engineering Guangdong Laboratory (Zhanjiang) Zhanjiang Guangdong China

**Keywords:** gel texture, microstructure, *Nemipterus virgatus* surimi, vegetable oils

## Abstract

To enhance the quality and flavor of surimi‐based products, we investigated the effects of vegetable oils (peanut, soybean, corn, coconut, olive, and safflower seed oils) on the texture, water‐holding capacity (WHC), microstructure, and flavor of the *Nemipterus virgatus* surimi gel. The results showed that 6 kinds of vegetable oils could improve the whiteness and flavor of gels. However, peanut, olive, and coconut oils enriching oleic acid or lauric acid were easy to accumulate with an average diameter of more than 0.15 μm. Thus, the gel with the oil showed a loose network structures with large cavities, and the texture was deteriorated, accompanied by decreased WHC (*p* < .05). Compared with other vegetable oils, soybean, corn and safflower seed oils enriching linoleic acid were emulsified with protein forming a stable interfacial protein film. The gel with the oil showed an increase in the WHC and bound water content. Furthermore, the oil droplets with an average diameter of less than 0.15 μm were evenly distributed in the gel matrix, and the gel exhibited dense network structures with small cavities and smooth surface. In general, soybean and safflower seed oils can be used as a potential additive to improve the quality and flavor of surimi‐based products.

## Graphical abstract

Vegetable oils can improve the whiteness and flavor of surimi gel. Surimi gels with soybean, corn, or safflower seed oils have a denser gel 3D network structure than those with other vegetable oils. High‐quality of surimi‐based products can be produced by adding vegetable oils that rich in polyunsaturated fatty acids.

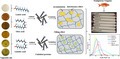

## 1 INTRODUCTION

Surimi is a functional ingredient used for surimi‐based products and produced by collecting meat, washing, dehydrating, and filtering (Singh et al., 2020). As a high‐protein food with high nutrient values, surimi‐based products have been widely accepted by consumers because of inexpensive protein source as well as unique gel properties (Mi et al., 2021). According to China fishery statistical yearbook, the processing capacity of surimi in 2019 was 1.394 million tons in China and accounted for 6.4% of the processed aquatic products, which was an increase of 64.4% over 2009 (Ministry of Agriculture, 2020). Thus, it shows a huge potential in the processing and utilization of aquatic products. During the washing stage of surimi, a large amount of valuable and nutritious fish lipids are removed for concentrating myofibrillar protein and reducing lipid oxidation on the storage, which is aimed at extending the shelf life and obtaining a stability quality of surimi (Jiao et al., 2019; Zhang et al., 2017). However, fish lipids are essential for maintaining the texture and giving unique flavors for surimi‐based products (Jiao et al., 2019; Pietrowski et al., 2012). Meanwhile, lipid deficiency will cause a poor texture and off‐flavor of surimi‐based products, which severely restricted the widespread use of surimi‐based products (Choi et al., 2010; Jiao et al., 2019).

To improve the quality and flavor of surimi‐based products, exogenous lipids are often added as texture modifiers, color enhancers, and processing aids during the processing of surimi‐based products (Liu et al., 2019). Generally, animal fats are rich in long‐chain saturated fatty acids and cholesterol, which greatly increase the incidence of obesity, hypertension, cardiovascular disease and coronary heart disease (Paneras & Bloukas, 1994; Shi et al., 2014). Moreover, the increasing attention to healthy food, consumers are more inclined to choose new surimi‐based products with less or no animal fat (Shi et al., 2014). Compared with the animal fat, vegetable oils are cholesterol free and rich in unsaturated fatty acids, and thus they are often used as a substitute for animal fat to improve the quality and flavor of surimi‐based products (Chang et al., 2015; Gani et al., 2018; Zhou et al., 2017). However, different vegetable oils are different in the composition and content of fatty acids, which have different effects on the quality and flavor of surimi‐based products. The camellia oil enriching oleic acid can effectively improve the whiteness, texture, and sensory properties of the white croaker surimi gel (Zhou et al., 2017), while the virgin coconut oil enriching lauric acid can significantly reduce the texture and WHC of the croaker surimi gel (Gani et al., 2018). Mi et al. (2017) also found that soybean, flaxseed, and perilla seed oils showed different effects on the whiteness, WHC, texture, and microstructure of grass carp surimi gel. And when perilla seed oil was added to 3%, the grass surimi gel showed a denser three‐dimensional gel network structure than that of soybean and flaxseed oils. In addition, vegetable oils (soybean, peanut, corn, and rap oil) also could improve the whiteness and sensory properties of the silver carp surimi gel, but the surimi gels containing peanut oil showed higher breaking force and lower expressible water than surimi gels with other oils (Shi et al., 2014). Therefore, different types of vegetable oils have different effects on the quality of surimi‐based products. For specific surimi and surimi‐based products, it is necessary to screen the best type of vegetable oils to improve the quality and flavor of surimi‐based products.

At present, the common vegetable oils mainly include peanut, soybean, corn, coconut, olive, and safflower seed oils, etc. Among them, peanut, soybean, and corn oils are commonly cooking vegetable oils in China (Shi et al., 2014). And the coconut, olive, and safflower seed oils are common functional vegetable oils. Additionally, these vegetable oils are rich in unsaturated fatty acids or medium‐chain saturated fatty acids, which plays an important role in reducing plasma high‐density lipids and cholesterol (Liu et al., 1991; Shi et al., 2014). Meanwhile, the *Nemipterus virgatus* is an important material for surimi‐based products because of high‐content protein and strong gel properties (Fang et al., 2021). In addition, *N. virgatus* is an important economic fish species in the South China Sea with high edible value and rich nutrients. China collected approximately 329.19 thousand tons of *N. virgatus* in 2019, showing a great potential for resource development (Ministry of Agriculture, 2020). At present, the processing methods of *N. virgatus* mainly includes surimi and surimi‐based products, frozen processing, dried fish products, and fish oil. Among them, surimi and surimi‐based products are the most widely processed and used. And there are few studies on the effect of vegetable oils on the quality and flavor of surimi‐based products. In particular, polyunsaturated fatty acids were more conducive to forming a stable system with protein emulsification, while medium‐chain, monounsaturated, or saturated fatty acids were not beneficial to forming a stable system with protein emulsification (Zheng et al., 2021). Thus, we hypothesized that high quality of surimi‐based products can be produced by adding vegetable oils that are rich in polyunsaturated fatty acids. Therefore, the objective of the study was to compare the effects of different vegetable oils on the quality and flavor, and to screen out the best type of oil to improve the quality and flavor of the *N. virgatus* surimi gel. The result might help to elucidate the relationship between the type and composition of vegetable oils and the quality of surimi gel, which could provide guidance for the development of new surimi‐based products.

## 2 MATERIALS AND METHODS

### 2.1 Materials and chemical reagents

Frozen *N. virgatus* surimi (AAA‐grade, moisture content: 73.96 ± 0.26%) was purchased from Fenghua Food Co., Ltd., (Beihai, China) and stored at −20℃. Peanut, coil and soybean oils were purchased from Yihai Kerry Food Marketing Co., Ltd., (Shanghai, China). Coconut oil was purchased from Panchen Trading Co., Ltd., (Shanghai, China). Olive oil was purchased from Pinwo Food Co., Ltd., (Shanghai, China). Safflower seed oil was purchased from COFCO Tayuan Honghua Co., Ltd., (Xinjiang, China). Tissue‐Tek O.C.T. was purchased from Sakura Finetek Japan Co., Ltd., (Tokyo, Japan). Tween 20 was purchased from Aladdin Biochemical Technology Co., Ltd., (Shanghai, China). Linoleic acid (≥95%, GC) was purchased from Macklin Biochemical Co., Ltd., (Shanghai, China). The rest of chemical reagents used in the experiments were analytical grade and purchased from Chemical Reagent Factory (Guangzhou, China).

### 2.2 Preparation of composite surimi gel

After thawing at 4℃ overnight, surimi was cut into small pieces. Salt (2.5 g/100 g surimi) was added into surimi and chopped at the speed of 2100 rpm for 2 min in a Stephan vertical vacuum cutter (Model UM 5, Stephan Machinery Co., Hameln, Germany). Subsequently, 2 ml/100 g of vegetable oils (peanut, soybean, corn, coconut, olive, and safflower seed oils) were added into surimi pastes and the final moisture content was adjusted to 80% with ice water, chopping at the same speed for 3 min. And surimi gel without vegetable oils was used as the control. After eliminating the air pockets, surimi was poured into plastic casing with the diameter of 2.5 cm and sealed at both ends. During chopping, water was used as cooling medium to keep the sample temperature below 8℃. Finally, samples were set at 40℃ for 30 min and then hotwater bath at 90℃ for 20 min. After water bath heating, samples were immediately put in ice water and then stored at 4℃ (Gani & Benjakul, 2018; Yan et al., 2020; Zhou et al., 2017).

### 2.3 Whiteness evaluation

After equilibrating at room temperature, the surimi gel was cut into thin slices. The L* (lightness), a* (redness/greenness), and b* (yellowness/blueness) were measured by using a spectrophotometer (Model NS800, 3NH technology Co., Ltd., Shenzhen, China). Each sample was measured in replications of five and the average value was taken. The whiteness of gel was calculated by the following equation (Fan et al., 2020; Meng et al., 2021; Eq. (1)):

(1) $$\text{Whiteness}=100‐\sqrt{{\left(100‐L^{*}\right)}^{2}+a^{*2}+b^{*2}}$$

### 2.4 Texture properties of gel

Texture properties of gel was performed by applying texture properties analysis (TPA) measurement mode and gel strength measurement mode of texture analyzer (Model TA.XT plusC, STab. Micro System, Ltd., Surrey, Britain), and probe models were P/0.5S spherical plunger probe and P/0.5 flat plunger probe, respectively (Jiao et al., 2019). Briefly, after equilibrating at room temperature, the plunger probe was pressed into the cross‐section of sample perpendicularly. Other test parameters were as follows: pretest, test, and post‐test speed 1.00 mm/s; trigger force 5 g and compression strain 50%. Subsequently, TPA parameters (hardness, adhesiveness, springiness, cohesiveness, gumminess, chewiness, and resilience) were calculated by Texture Expert software version 1.22.

### 2.5 Water‐holding capacity (WHC)

The WHC of gel was measured according to the method (Zhou et al., 2017). After cutting into small pieces, approximately 3.0 g of gel samples were weighed accurately (*M*_1_) and wrapped with two filter papers. Subsequently, gel samples were centrifuged (J‐26sxp; Avanti, Beckman, USA) at 10,000 rpm for 10 min, and weighed again (*M*_2_). The WHC was calculated based on the following equation (Eq. (2)):

(2) $$\text{WHC}/\%=\frac{M_{2}}{M_{1}}\times 100$$

### 2.6 Cooking loss rate (CLR)

The CLR was performed based on the method (Liu et al., 2021) with some modifications. A 5 × 15 × 15 mm of gel sample was weighed accurately (*G*_1_) and put into a cooking bag. After the gel sample was heated at 90℃ for 20 min, the liquid on the surface of gel sample was dried by using filter papers, and weighed (*G*_2_) again. The CLR was calculated by the following equation (Eq. (3)):

(3) $$\text{CLR}/\%=\frac{G_{1}‐G_{2}}{G_{1}}\times 100$$

### 2.7 Moisture distribution and composition

Surimi gel was cut into small cylinders (40 × 15 × 15 mm^3^) and placed in a low‐field nuclear magnetic resonance (LF‐NMR) tube with a diameter of 40 mm. The transverse relaxation time (*T*_2_) was determined using a LF‐NMR analyzer (NMI20‐060H‐I; Niumag Co., Ltd., Suzhou, China). The measurement parameters were set as: SFI = 22 MHz, *τ* = 400 μs, NS = 8, TR = 6 s, EchoCount = 1500. Then, each peak area in integral spectrum of the *T*_2_ was accumulated for calculation moisture distribution and composition on surimi gels (Jiao et al., 2019).

### 2.8 Light microscopic images analysis

Surimi gels were dehydrated by 30% sucrose, embedded, and fixed with Tissue‐Tek O.C.T., and then samples were cut into 20‐μm‐thick slides by using a microtome (Leica CM1950, Leica Microsystems Ins., Germany). Then samples were respectively dyed with 1% bromophenol blue solution (protein dye) and 0.1% Sudan IV dye solution (fat dye) (Liu et al., 2019; Zhuang et al., 2016). The distributions of oil droplets on surimi gels were observed using an Olympus microscope (CKX41, Olympus Optical Co., Ltd. Tokyo, Japan). ImageJ 1.8.0.17 (version 1.52 t) software was used to measure the droplet diameter and then draw the droplet diameter distribution image.

### 2.9 Scanning electron microscopy

Microstructure of gel was observed following the method described by Feng et al. (2018) with some modifications. Briefly, surimi gels were cut into thick slices, fixed by glutaraldehyde solution, washed by phosphate buffer, successively dehydrated by ethanol, degreased by chloroform, replaced by tert‐butanol, and freeze‐dried. At an acceleration voltage of 8 kV, the microstructure of gel was observed using an *SEM* (7610F; Japan Electronics Co., Ltd., Tokyo, Japan) at a magnification of 15,000×.

### 2.10 Lipid oxidation of surimi gel

Thiobarbituric acid reaction substances (TBARS) were used to evaluate the degree of lipid oxidation in surimi gels. Malondialdehyde (MDA) content in the gels was determined according to the method described by Pietrowski et al., (2011) and Aheto et al. (2020) with some modifications. Surimi gels (5.0 g) were mixed with 7.5% trichloroacetic acid (50 ml) containing 0.1% EDTA and heated at 50°C for 30 min. The mixtures were filtered using two layers of filter paper. The filtrate (2 ml) was mixed with 0.02 M thiobarbituric acid solution (2 ml) and heated at 90°C for 30 min. After cooling, the absorbance was measured at 532 nm using a UV‐Vis spectrophotometer (Cintra 1010; GBC Scientific Equipment Pty Ltd, Sydney, Australia). The MDA standard curve was formulated from 1,1,3,3‐tetraethoxypropane, and TBARS content was expressed as the mass of MDA per kilogram of gel (mg/kg).

### 2.11 Lipoxidase activity of surimi gel

Lipoxygenase activity was determined according to a previously described method (Gao et al., 2020; Huang et al., 2017) with some modifications. Briefly, 50 mM phosphate buffer solution (pH 7.4, 1.0 mM dithiothreitol, and 1.0 mM EDTA) was mixed with gel samples at a ratio of 4:1 (v/w), and then homogenized at 15,000 rpm in an ice bath for 1 min. After filtering through four layers of gauze, the mixtures were centrifuged at 10,000 ***g*** for 30 min. The supernatant was regarded as a lipoxygenase crude enzyme solution for further analysis. For the substrate solution, linoleic acid (140 µl) was mixed with Tween 20 (180 µl) and emulsified in 10 ml of deoxygenated redistilled water. The pH of the mixtures was adjusted to 9.0, with 2 M NaOH, and diluted with deoxygenated redistilled water to 50 ml. The activity of lipoxygenase was determined by measuring the increase in the absorbance of the substrate solution at a wavelength of 234 nm using a UV‐Vis spectrophotometer (Cintra 1010; GBC Scientific Equipment Pty Ltd, Sydney, Australia) at room temperature (25°C). A total of 0.1 ml of enzyme solution was added to a quartz cell containing 50 mM citric acid solution (2.9 ml, pH 5.5) and 0.2 ml linoleic acid substrate solution quickly, and the increase in absorbance was recorded within 1 min. The unit of lipoxygenase activity was U/g, where U was defined as an increase in absorbance of 0.001 per minute. The control consisted of 0.2 ml of linoleic acid substrate solution and 3.0 ml of citric acid buffer solution. Each sample was measured five times, and the average values were calculated.

### 2.12 Statistical analysis

All experiments were independently implemented in triplicate or more with many different samples. Statistical analysis (ANOVA and Duncan's Multiple Range test) was analyzed by using SPSS statistic 19.0 software (SPSS Inc., Chicago, IL, USA). All figures were expressed in the form of mean ± standard deviations (*SD*).

## 3 RESULTS AND DISCUSSION

### 3.1 Whiteness analysis of surimi gel

**FIGURE 1:**
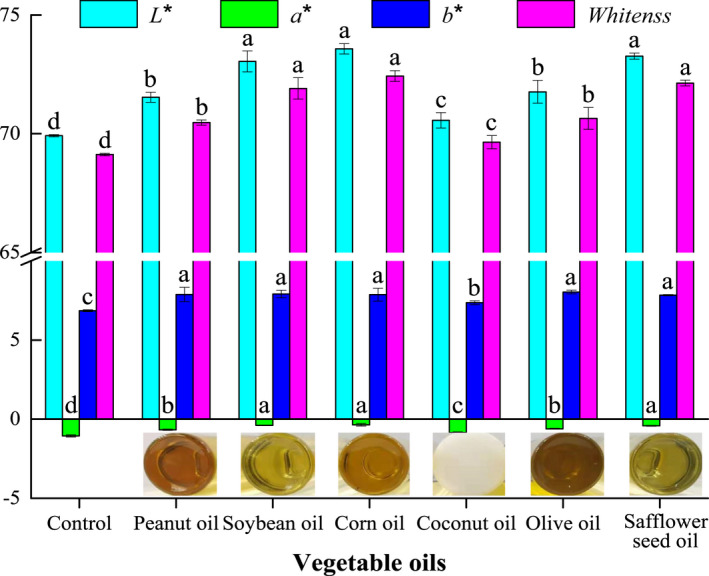
Effects of vegetable oils on the whiteness of the *N. virgatus* surimi gel

Whiteness is an important parameter that reflects the color and quality of surimi gel, and it can directly determine the preference of consumer (Alipour et al., 2018; Liu et al., 2019). The change in whiteness depends on the changes in three‐dimensional network structure of surimi gel, which closely relates to denaturation, aggregation, and cross‐linking of myofibrillar protein (Bao et al., 2018; Feng et al., 2018; Shi et al., 2014). As shown in Figure 1, after adding 2 ml/100 g vegetable oils, the L*, a* and b* value of surimi gel increased (*p* < .05), and thus the whiteness of surimi gel increased (*p* < .05). Additionally, the gel containing different vegetable oils showed a significant difference in the whiteness. And the addition of soybean, corn, and safflower seed oils had a greater effect on the whiteness of gel than other vegetable oils. These demonstrated that vegetable oils could affect interactions between protein molecules and change the network structure of surimi gel. Meanwhile, different vegetable oils showed different effects on the network structure of gel due to the different compositions and contents of fatty acids. Moreover, because of soybean, corn, and safflower seed oils with a light and bright color, surimi gel with these oils could thus enhance light‐scattering and light‐reflecting ability to improve their whiteness (Pietrowski et al., 2011; Shi et al., 2014). Similarly, compared with other vegetable oils, peanut and olive oils had a dark and dim color, and coconut oil was easy to solidify at low temperature, which showed poor improvement on the whiteness of surimi gel (Motamedzadegan et al., 2020). Therefore, vegetable oils with the light and bright color can significantly enhance the whiteness of the *N. virgatus* surimi gel.

### 3.2 Texture analysis of surimi gel

The texture is a fundamental indicator that can determine the gelling ability of surimi during heating, and it mainly depends on the formation and stability of the three‐dimensional network structure (Chang et al., 2015). The texture of the *N. virgatus* surimi gel with vegetable oils is shown in Table 1. After adding 2 ml/100 g vegetable oils, there was a significant decrease in the gel strength, rupture strength, hardness, cohesiveness, and chewiness (*p* < .05). But compared with other vegetable oils, the surimi gel with soybean, corn or safflower seed oils showed a stronger texture, followed by the surimi gel with peanut and olive oils, and the surimi gel with coconut oil showed the weakest texture (*p* < .05). It demonstrated that vegetable oils were adverse for the texture of surimi gel, and the effect might depend on the composition and content of fatty acid in vegetable oils. It is in accordance with the result published by Mi et al. (2017), who found that the grass surimi gel with perilla seed oil showed the gel network structures denser than those with soybean or flaxseed oils. Gani et al. (2018) also found that coconut oil enriching lauric acid significantly deteriorated the texture of the croaker surimi gel.

**TABLE 1:** Effects of vegetable oils on texture of the *Nemipterus virgatus* surimi gel

Vegetable oils	Control	Peanut oil	Soybean oil	Corn oil	Coconut oil	Olive oil	Safflower seed oil
Gel strength (*N*)	4.934 ± 0.041^a^	3.856 ± 0.041^e^	4.332 ± 0.112^c^	4.027 ± 0.061^d^	3.672 ± 0.103^f^	3.850 ± 0.095^e^	4.605 ± 0.085^b^
Rupture strength (*N*)	13.558 ± 0.099^a^	12.567 ± 0.094^e^	12.914 ± 0.076^c^	12.751 ± 0.063^d^	11.991 ± 0.208^f^	12.513 ± 0.104^e^	13.130 ± 0.081^b^
Hardness (*N*)	10.961 ± 0.086^a^	9.892 ± 0.106^d^	10.342 ± 0.152^c^	10.303 ± 0.159^c^	9.620 ± 0.079^e^	9.905 ± 0.128^d^	10.621 ± 0.094^b^
Adhesiveness (g·s)	2.102 ± 0.106^a^	1.939 ± 0.006^c^	2.080 ± 0.093^a^	2.099 ± 0.098^a^	1.769 ± 0.071^d^	1.951 ± 0.088^c^	2.060 ± 0.059^a^
Springiness	0.588 ± 0.008^c^	0.621 ± 0.008^a^	0.607 ± 0.008^b^	0.604 ± 0.008^b^	0.626 ± 0.006^a^	0.622 ± 0.007^a^	0.607 ± 0.004^b^
Cohesiveness	0.591 ± 0.005^a^	0.532 ± 0.011^d^	0.558 ± 0.007^c^	0.565 ± 0.009^c^	0.517 ± 0.007^e^	0.531 ± 0.007^d^	0.577 ± 0.005^b^
Gumminess	5.991 ± 0.104^a^	5.839 ± 0.167^a^	5.891 ± 0.069^a^	5.850 ± 0.127^a^	5.503 ± 0.115^b^	5.889 ± 0.100^a^	5.893 ± 0.079^a^
Chewiness	4.021 ± 0.145^a^	3.376 ± 0.085^c^	3.608 ± 0.103^b^	3.650 ± 0.093^b^	3.049 ± 0.101^d^	3.387 ± 0.123^c^	3.676 ± 0.091^b^
Resilience	0.221 ± 0.003^b^	0.242 ± 0.005^a^	0.241 ± 0.005^a^	0.239 ± 0.002^a^	0.238 ± 0.005^a^	0.238 ± 0.004^a^	0.239 ± 0.007^a^

Note

The data are expressed in the form of mean ± standard deviations (*n* = 5). Different letters within the same row indicate significant differences (*p* < .05) between mean values.

However, there is a positive correlation between the texture and protein content of surimi gel (Chang et al., 2015). Myofibrillar proteins are the main protein that form the three‐dimensional network structure of surimi gel. The addition of vegetable oils into surimi will lead to a relative decrease in the protein content of surimi gel (Chang et al., 2015). Thus, the gel containing vegetable oils showed a network structure looser than those without vegetable oils. However, camellia oil enriching oleic acid could effectively improve the texture of the white croaker surimi gels (Zhou et al., 2017), while coconut oil enriching lauric acid could significantly reduce the texture of the croaker surimi gels. In addition, vegetable oils containing more polyunsaturated fatty acids are more conducive to forming a stable system with protein emulsification (Zheng et al., 2021). However, the oil containing more medium‐chain, monounsaturated or saturated fatty acids are not beneficial to forming a stable system with protein emulsification (Zheng et al., 2021). Therefore, it is soybean, corn, and safflower seed oils that are rich in polyunsaturated fatty acids. Thus, surimi gel with these vegetable oils show a texture stronger than that with other vegetable oils.

### 3.3 The WHC and CLR analysis of surimi gel

**FIGURE 2:**
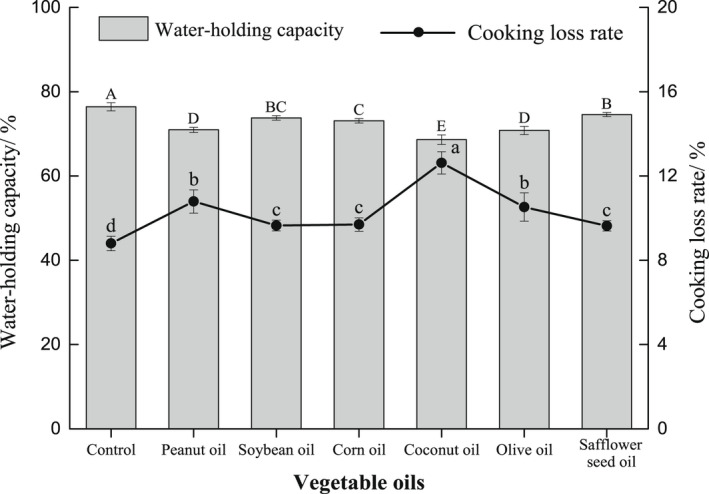
Effects of vegetable oils on water‐holding capacity and cooking loss rate of the *N. virgatus* surimi gel

During the gelation process of surimi, the high‐level structure of myofibrillar protein becomes weak by heating, and then forms a three‐dimensional network structure to trap free water in the gel matrix (Bao et al., 2018; Feng et al., 2018; Shi et al., 2014). Additionally, the CLR is similar to the WHC, which can reflect the ability of surimi gel to retain water. The surimi gel with high WHC and low CLR can trap a great deal of water, and is less likely to lose water during cooking (Bao et al., 2018; Ma et al., 2015). As shown in Figure 2, after adding 2 ml/100 g vegetable oils, the WHC of the *N. virgatus* surimi gel decreased (*p* < .05), and the CLR increased (*p* < .05). But compared with other vegetable oils, the surimi gel with soybean, corn, or safflower seed oils showed the highest WHC and lowest CLR, followed by the surimi gel with peanut and olive oils, and the surimi gel with coconut oil showed the lowest WHC and highest CLR (*p* < .05). These results also proved that different vegetable oils showed different effects on the network structure of surimi gel, and the oils enriching polyunsaturated fatty acids would be beneficial for surimi gel to retain water.

Vegetable oils can interfere in interaction between protein molecules by increasing the distances between protein molecules (Liu et al., 2019; Mourtzinos & Kiosseoglou, 2005; Yan et al., 2020). Thus, vegetable oils were adverse to the density and uniformity of network structure in surimi gel. And the damage to network structure will be increasingly great with the size of oil droplets getting large. Moreover, the hydrophobic long‐chain alkyl group of fatty acid also may occupy the original voids of water molecules in surimi gel (Zhou et al., 2017). These characteristics resulted in a decrease in the WHC and an increase in the CLR of surimi gel during the heating process. However, the effect of vegetable oils on the network structure of surimi gel may depend on the carbon chain length and saturation of fatty acids. Medium‐chain, monounsaturated, or saturated fatty acids were emulsified with protein to form an unstable system, which cannot inhibit the aggregation of oil droplets and the adjacent oil droplets is easy to gather and collapse (Zheng et al., 2021). On the contrary, polyunsaturated fatty acids were emulsified with protein to form a stable system, so that they can prevent oil droplets from aggregation and collapsing (Zheng et al., 2021). Consequently, soybean, corn and safflower seed oils enriching linoleic acid (Table A1) can evenly distributed in the gel matrix, while peanut, olive and coconut oils enriching oleic acid or lauric acid (Table A1) are tend to accumulate in the gel matrix. Therefore, compared with other vegetable oils, soybean, corn, and safflower seed oils had little damage to the network structure of surimi gel, and thus the gel with the oils exhibited a high WHC and a low CLR.

### 3.4 Moisture distribution and composition

Effects of vegetable oils on moisture composition of the *N. virgatus* surimi gel is shown in Table 2. After adding 2 ml/100 g vegetable oils, the content of bound water in the *N. virgatus* surimi gel decreased (*p* < .05), and the content of free water increased (*p* < .05). In addition, surimi gel containing coconut oil had the lowest bound water content and the highest free water content (*p* < .05). However, there is no significant difference in the content of bound water, immobilized water, and free water in the gel containing other vegetable oils (*p* > .05). Vegetable oils can disrupt the gel's network structure by increasing the distance between protein molecules (Liu et al., 2019). Surimi gel with a loose network structure cannot fully lock in water, which causes bound water and immobilized water to move easily, and then discharges from tissue structure in the form of free water (Jiao et al., 2019). Therefore, the gel containing vegetable oils showed the decrease in the WHC and increase in the CLR. Compared with other vegetable oils, coconut oil enriching lauric acid tends to accumulate easily, and form large oil droplets at the low temperature. Therefore, surimi gel containing coconut oil showed a loose network structure with the highest content of free water and the lowest content of bound water.

**TABLE 2:** Effects of vegetable oils on moisture composition of the *N. virgatus* surimi gel

Vegetable oils	Bound water/%	Immobilized water/%	Free water/%
Control	3.760 ± 0.118^a^	96.079 ± 0.126^b^	0.161 ± 0.009^c^
Peanut oil	3.341 ± 0.119^b^	96.307 ± 0.129^b^	0.352 ± 0.013^b^
Soybean oil	3.440 ± 0.135^b^	96.228 ± 0.131^b^	0.332 ± 0.016^b^
Corn oil	3.478 ± 0.079^b^	96.185 ± 0.066^b^	0.338 ± 0.012^b^
Coconut oil	2.918 ± 0.192^c^	96.608 ± 0.182^a^	0.474 ± 0.012^a^
Olive oil	3.360 ± 0.130^b^	96.285 ± 0.140^b^	0.355 ± 0.010^b^
Safflower seed oil	3.343 ± 0.110^b^	96.323 ± 0.105^b^	0.334 ± 0.014^b^

Note

The data are expressed in the form of mean ± standard deviations (*n* = 5). Different letters within the same column indicate significant differences (*p* < .05) between mean values.

### 3.5 Oil droplet diameter size distribution

**FIGURE 3:**
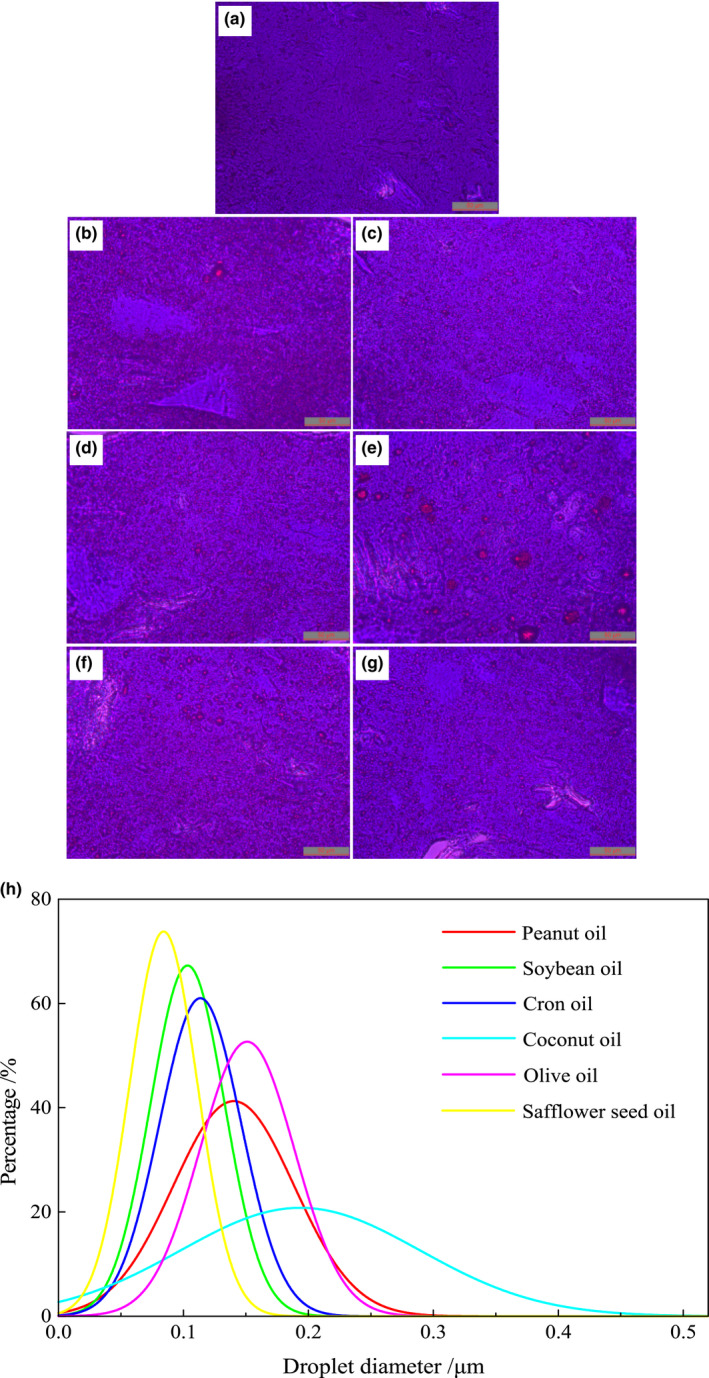
Effects of vegetable oils on the oil droplet distribution (×400) of the *N. virgatus* surimi gel. (a): control; (b‐g): the surimi gel containing peanut, soybean, corn, coconut, olive, or safflower seed oils, respectively; (h): vegetable oils droplets diameter distribution image

Optical microscope image can truly identify the distribution and aggregation of oil droplets in the gel matrix. As shown in Figure 3, there are no traces of oil droplets in the control, and the surface of the *N. virgatus* surimi gel was relatively smooth, (Figure 3a). However, after adding 2 ml/100 g vegetable oils, the regular traces of oil droplets could be clearly observed on the surface of surimi gel. Because of difference in the composition and content of fatty acids, different emulsifying effects between vegetable oils and proteins lead oil droplets to distribute and aggregate in difference (Zheng et al., 2021). Medium‐chain, monounsaturated or saturated fatty acids are emulsified with protein to form an unstable system (Zheng et al., 2021), which fail to prevent adjacent oil droplets from accumulating into large oil droplets. Thus, peanut and olive oils enriching oleic acid were unevenly distributed in the gel matrix (Figure 3b and f). The diameter of oil droplets was mainly in the range of 0.10–0.20 μm (Figure 3h). Especially, there were coconut oil droplets with an average diameter >0.20 μm in the gel matrix (Figure 3e). Furthermore, oil droplets merge into larger oil droplets to decrease the interface energy, which eventually interferes with the formation of network structure during heating (Liu et al., 2019). And the formed large oil droplets further promote oil droplets to accumulate. However, soybean, corn, and safflower seed oils with an average diameter of <0.15 μm were evenly distributed in the gel matrix (Figure 3c, d and g), which may be attributed to enriching linoleic acid. There is an increase in the surface area of oil droplets in the same region (Liu et al., 2019). Based on these results, we hypothesized that the oil enriching polyunsaturated fatty acids was evenly distributed in the gel matrix, which showed little damage to the network structure of surimi gel.

### 3.6 Microstructure of surimi gel

**FIGURE 4:**
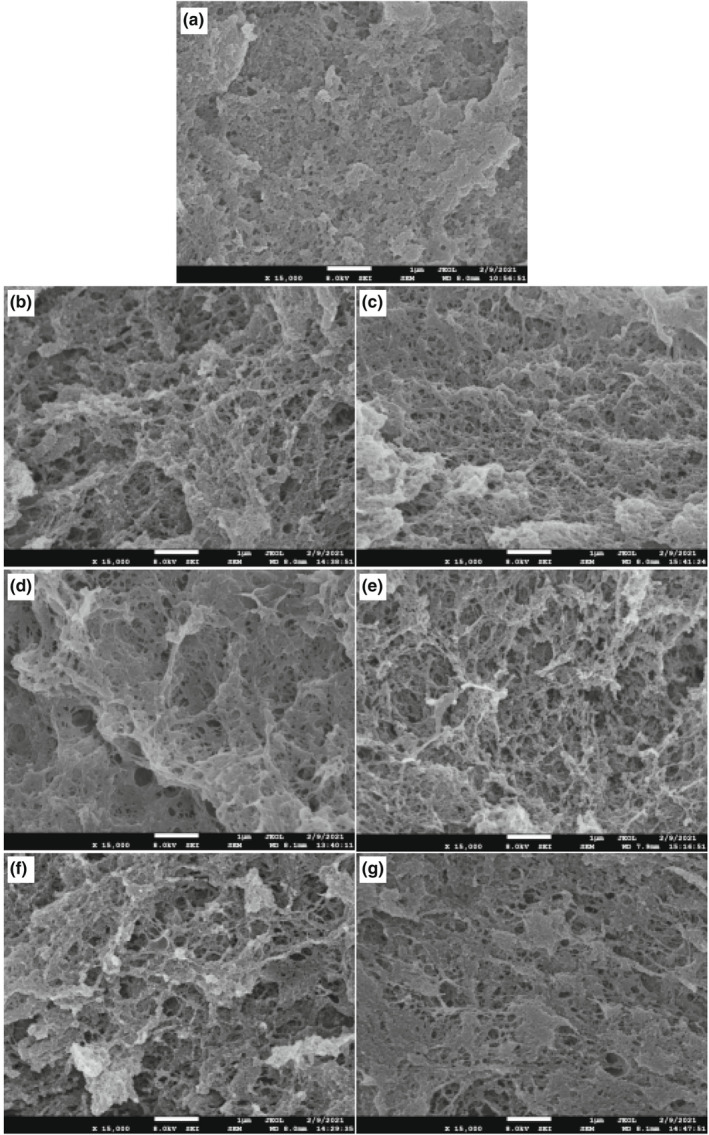
Effects of vegetable oils on scanning electron microscope photograph (×15,000) of the *N. virgatus* surimi gel. (a): control; (b‐g): the surimi gel containing peanut, soybean, corn, coconut, olive, or safflower seed oil, respectively

The quality of surimi‐based products is closely related to the microstructure of surimi gel, which can directly reflect the gelation ability of surimi during heating. The formation of microstructure depends on the orderly aggregation of myofibrillar proteins and the interaction between protein molecules (Bao et al., 2018; Feng et al., 2018; Shi et al., 2014). As shown in Figure 4, after adding 2 ml/100 g vegetable oils, the surimi gels with soybean, corn or safflower seed oils exhibited a dense three‐dimensional network structure with small cavities and smooth surface, while the gel with peanut, olive, or coconut oils exhibited a loose three‐dimensional network structure. These results further confirmed that the effect of different vegetable oils on the quality of gel mainly depended on the fatty acid composition and content of vegetable oils. And the results were in accordance with the texture, WHC, and CLR of the gel (Table 1 and Figure 2). Vegetable oils enriching monounsaturated or saturated fatty acids was more unfavorable to the texture of surimi gel than those enriching polyunsaturated fatty acids (Zheng et al., 2021). The emulsification ability of monounsaturated and saturated fatty acids with protein is not as good as that of polyunsaturated fatty acids (Zheng et al., 2021). And medium‐chain fatty acids tend to gather and form large oil droplets at low temperatures. Thus, vegetable oils enriching medium‐chain, monounsaturated or saturated fatty acids caused a great damage to the network structure of surimi gel. Furthermore, vegetable oils may also play a role, as filler particles occupy the void spaces in the network structure of surimi gel (Gani et al., 2018). Therefore, compared with other vegetable oils, surimi gel containing soybean, corn or safflower seed oils exhibited a dense and uniform three‐dimensional network structure.

### 3.7 Lipid oxidation and lipoxygenase activity of surimi gel

**FIGURE 5:**
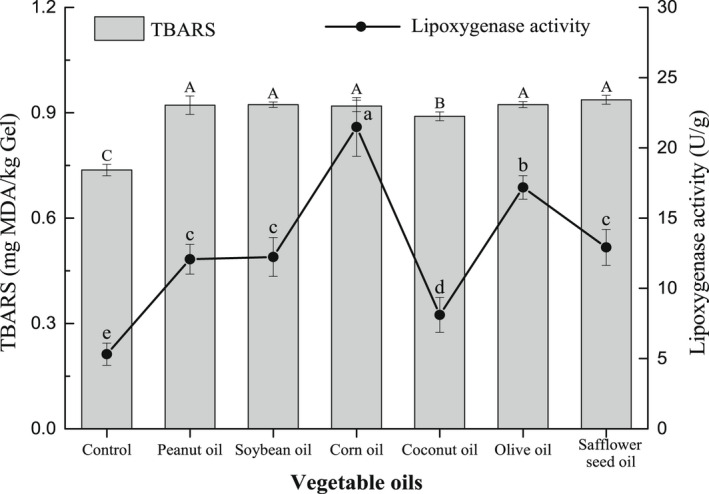
Effect of vegetable oils on the TBARS content and lipoxygenase activity of the *N. virgatus* surimi gel

Malondialdehyde (MDA) is a mainly secondary oxidation product that can be used to evaluate the degree of oxidation and rancidity (Pietrowski et al., 2011). The TBARS content is an important index to measure the content of MDA. The TBARS content increased with the increase in the MDA content, which indicates that the oxidation degree of oil was increased. As shown in Figure 5, the TBARS content of the *N. virgatus* surimi gel was 0.74 mg/kg, and the TBARS content of surimi gel increased after adding vegetable oils. But compared with other vegetable oils, the TBARS content was low in the surimi gel containing coconut oil. It demonstrated that coconut oil in surimi gel was not easy to be oxidized and decomposed to produce MDA. The fatty acid composition of coconut oil is mainly saturated fatty acids such as lauric acid, which is difficult to produce MDA during heating. However, other vegetable oils are rich in oleic acid and linoleic acid, which are easily oxidized to produce MDA. Thus, the TBARS content was high in the surimi gel containing these vegetable oils. In this study, the TBARS content was <1.2 mg/kg gel in the *N. virgatus* surimi gel with vegetable oils (Figure 5). Aheto et al. (2020) reported that the maximum TBARS content was 4.75 mg MDA/kg in the dried pork products. Berruga et al. (2005) also pointed out that the acceptable levels of the TBARS content was <4.2–7.5 mg MDA/kg in meat products. Surimi gel containing vegetable oils in such TBARS content was far below the acceptable range. Therefore, vegetable oils can be used as processing aids to add into surimi gel, but necessary antioxidant measures should be taken into consideration to improve the storage stability of surimi gel.

Under the specific catalysis of lipoxygenase, unsaturated fatty acids are oxidized to produce hydroperoxides (Gao et al., 2020; Huang et al., 2017). These hydroperoxides are further decomposed into various volatile compounds including aldehydes, ketone, and their alcoholic counterparts (Mandal et al., 2014). And byproducts of oil oxidation may contribute to volatile flavor components in food system (Kwan & Davidovpardo, 2018; Shi et al., 2014). Effect of vegetable oils on lipoxygenase activity of the *N. virgatus* surimi gel was shown in Figure 5. After adding 2 ml/100 g vegetable oils, the activity of lipoxygenase in surimi gel was enhanced, which showed that vegetable oils could give unique flavors to the surimi gel. Moreover, the activity of lipoxygenase was the highest in the surimi gel containing corn oil and the lowest in the surimi gel containing coconut oil. It demonstrated that vegetable oils caused different effects to the activity of lipoxygenase, and then gave unique flavors to the surimi gel. Moreover, the degree of oil oxidation was positively correlated with the activity of lipoxygenase, which might contribute to the flavor in the early stage of surimi gel (Mandal et al., 2014). Yet it would definitely result in an off‐flavor surimi gel in long‐term storage. Therefore, compared with other vegetable oils, soybean and safflower seed oils can not only improve the quality of surimi gel but also give unique flavors to the surimi gel.

### 3.8 Correlation analysis of surimi gel

**FIGURE 6:**
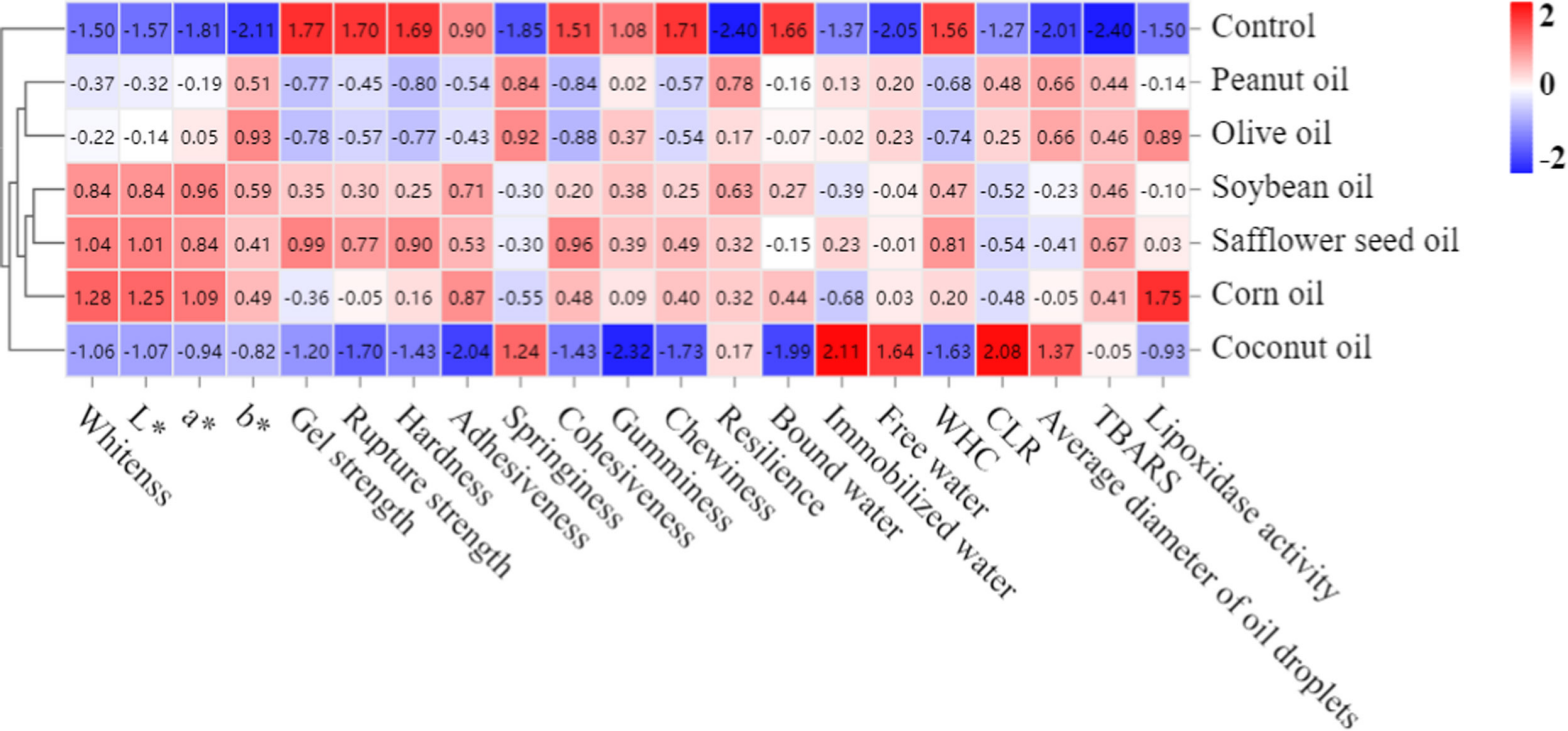
Effect of vegetable oils on the dynamic heatmap analysis of the *N. virgatus* surimi gel

To further explore the effects of vegetable oils on the dynamic heatmap analysis of the *N. virgatus* surimi gel, we investigated the dynamic heatmap analysis. As shown in Figure 6, based on the differences in the control and gels with vegetable oils, the gels were divided into four categories: type I (control), type II (the gel with coconut oil), type III (the gel with peanut or olive oils), and type IV (the gel with corn, soybean, or safflower seed oils). In addition, type I shows a strong negative correlation with whiteness, *L* ^*^, *a* ^*^, *b* ^*^, and lipoxidase activity, but other types show a weak negative correlation with these and even positive. which shows that vegetable oils can improve the whiteness and give unique flavors to the surimi gel. Type II and type III show a positive correlation with springiness, resilience, immobilized water, free water, CLR, and average diameter of oil droplets, and show a negative relationship with gel strength, rupture strength, hardness, adhesiveness, cohesiveness, chewiness, and WHC. However, compared with type II and type III, type IV is positively correlated with whiteness, *L* ^*^, *a* ^*^, *b* ^*^, hardness, adhesiveness, cohesiveness, chewiness, and WHC. Type IV also is negatively correlated with springiness, CLR, and average diameter of oil droplets. Therefore, due to soybean, corn, and safflower seed oils enriching polyunsaturated fatty acids, surimi gel with these vegetable oils thus shows a quality stronger than that with other vegetable oils.

## 4 CONCLUSION

The effect of vegetable oils on the quality and flavor of the *N. virgatus* surimi gel depends on the composition and content of fatty acid. Peanut, olive, and coconut oils enriching oleic acid or lauric acid were emulsified with protein forming an unstable system, which caused oil droplets to gather easily. An uneven distribution of oil droplets with a diameter in range of 0.10–0.20 μm was observed in the gel matrix, accompanied by obvious aggregation of oil droplets. Thus, the gel containing the olive, peanut, or coconut oils exhibited a loose three‐dimensional network with large cavities. However, soybean, corn, and safflower seed oils enriching linoleic acid were emulsified with protein to form a stable system, and the gels containing these oils show an increase in the whiteness, WHC, and bound water content. Furthermore, the oil droplets with an average diameter <0.15 μm were evenly distributed in the gel matrix, and the gel exhibited a dense three‐dimensional network with small cavities and smooth surface. The present results confirm our hypothesis that high quality of surimi‐based products can be produced by adding vegetable oils that are rich in polyunsaturated fatty acids. Yet, the way of adding vegetable oils needs to be changed to improve the texture of surimi gel.

## ACKNOWLEGEMENTS

The work was supported financially by the Innovation and Development Project about Marine Economy Demonstration of Zhanjiang City (Nos. XM‐202008‐01B1); the Southern Marine Science and Engineering Guangdong Laboratory (Zhanjiang) (Nos. ZJW‐2019–07); the Innovation Team Construction Project of Modern Agricultural Industry Technology System in Guangdong Province (Nos. 2021KJ150). The completion of this work is thanks to Professor Zhong‐ji QIAN's helps and guidances.

## CONFLICT OF INTERESTS

The author declares no conflict of interests.

## ETHICAL APPROVAL

This study does not involve any human or animal testing.

## Supporting information

Supplementary Material

## Data Availability

Research data are not shared.
