# Supplementary material for: Compare with different vegetable oils on the quality of the Nemipterus virgatus surimi gel
Source: Food Sci Nutr. 2022 Jun 2;10(9):2935–46. doi: 10.1002/fsn3.2889 (PMC9469861; doi:10.1002/fsn3.2889)
Supplement: Supplementary file 1 — Supplementary Material [file FSN3-10-2935-s001.docx]

**Supplementary Material**

**S 1. Method**

### **S1. Fatty acids composition of vegetable oils**

The fatty acid composition of vegetable oils was measured using gas chromatography of fatty acid methyl esters (FAMEs) (Lin et al., 2020). 0.5 g of vegetable oils were mixed with 5mL 0.5M sodium hydroxide-methanol solution, and methyl esterification was carried out at 60 °C for 1 h. After thorough reaction, 5 mL n-hexane was added for extracting FAMEs, and the supernatant was mixed with a small amount of anhydrous sodium sulfate and filtrated by 0.22 μm organic filter membranes. The FAMEs were injected into the gas chromatography mass spectrometry (GC-MS) (TQ8050NX, SHIMADZU Inc., Japan) with the auto-sampler. The heating procedure was set as follows: The initial temperature was set at 100 ℃ for 13 min, then increased to 180 ℃ at the speed of 10 ℃/min and kept for 6 min. Subsequently, the temperature at the speed of 1 ℃/min increased to 200 ℃ keeping 20 min, and then increased to 230 ℃ at the speed of 4 ℃/min which was kept at this temperature for 10.5 min. The injector temperature was set at 270 ℃ and the detector temperature was set at 280 ℃. Helium was supplied as carrier gas at the flow of 1.5 mL/min in a splitless mode. The ionization energy was 70 eV, and the mass scanning range was 35-550 amu. Qualitative was analyzed by matching each compound with the NIST 14 library database and mixed fatty acid methyl ester standard. And the relative percentage of fatty acids was calculated using the method of area normalization.

**S 2. Results**

**Table A1 The composition and relative content of fatty acids in vegetable oils**

| Fatty acids | Vegetable oils / % | | | | | |
| --- | --- | --- | --- | --- | --- | --- |
|  | Peanut oil | Soybean oil | Corn oil | Coconut oil | Olive oil | Safflower  seed oil |
| C 8:0 | — | — | — | 7.34±0.29^a^ | — | — |
| C 10:0 | — | — | — | 7.31±0.25^a^ | — | — |
| C 11:0 | — | — | — | 0.04±0.01^a^ | — | — |
| C 12:0 | — | — | — | 28.42±0.35^a^ | — | — |
| C 13:0 | — | — | — | 0.06±0.01^a^ | — | — |
| C 14:0 | 0.03±0.01^b^ | 0.08±0.01^b^ | 0.05±0.01^b^ | 19.62±0.30^a^ | 0.09±0.01^b^ | 0.15±0.01^b^ |
| C 16:0 | 11.39±0.12^e^ | 11.89±0.29^d^ | 18.67±0.18^a^ | 15.26±0.07^b^ | 13.07±0.03^c^ | 8.90±0.19^f^ |
| C 16:1 | 0.11±0.01^c^ | 0.08±0.01^c^ | 0.23±0.02^b^ | — | 1.17±0.02^a^ | 0.10±0.01^c^ |
| C 17:0 | 0.09±0.01^c^ | 0.11±0.01^b^ | 0.16±0.01^a^ | — | 0.09±0.01^c^ | 0.04±0.01^d^ |
| C 17:1 | 0.03±0.01^d^ | 0.05±0.01^c^ | 0.06±0.01^b^ | — | 0.12±0.01^a^ | — |
| C 18:0 | 5.62±0.05^c^ | 6.08±0.05^b^ | 4.94±0.16^d^ | 7.83±0.06^a^ | 4.73±0.01^e^ | 4.19±0.02^f^ |
| C 18:1 | 46.78±0.36^b^ | 32.87±0.3^c^ | 28.71±0.84^d^ | 1.69±0.02^f^ | 68.44±0.18^a^ | 18.25±0.02^e^ |
| C 18:2 | 27.47±0.46^d^ | 44.99±0.19^b^ | 43.68±0.41^c^ | 12.13±0.07^e^ | 11.12±0.14^f^ | 66.83±0.21^a^ |
| C 18:3 | 0.09±0.01^c^ | 3.20±0.33^a^ | 0.49±0.05^b^ | 0.12±0.01^c^ | — | 0.02±0.02^c^ |
| C 20:0 | 2.34±0.02^a^ | 0.04±0.01^e^ | 1.21±0.04^b^ | 0.17±0.01^d^ | 0.64±0.03^c^ | 0.65±0.01^c^ |
| C 20:1 | 1.28±0.02^a^ | 0.30±0.02^d^ | 0.99±0.04^b^ | — | 0.33±0.02^d^ | 0.39±0.01^c^ |
| C 22:0 | 4.72±0.14^a^ | 0.23±0.02^c^ | 0.44±0.01^b^ | — | 0.19±0.03^c^ | 0.41±0.01^b^ |
| C 22:1 | 0.05±0.02^b^ | 0.07±0.01^b^ | 0.38±0.01^a^ | — | — | 0.06±0.01^b^ |

Note: The data are expressed in the form of mean ± standard deviations (n=3). Different letters within the same row indicate significant differences (*P* < 0.05) between mean values, “—” means not detected.

**REFERENCES**

Lin, W. L., Han, Y. X., Liu, F. F., Huang, H., Li, L. H., Yang, S. L., et al. (2020). Effect of lipid on surimi gelation properties of the three major Chinese carp. *Journal of the Science of Food and Agriculture*, 100(13): 4671-4677. https://doi.org/[10.1002/JSFA.10414](https://schlr.cnki.net/Detail/doi/SJPDLAST/SJPD764AB0C9580209BA90C4BE09E66DBFF5)
